# The subgingival microbial community of feline periodontitis and gingivostomatitis: characterization and comparison between diseased and healthy cats

**DOI:** 10.1038/s41598-019-48852-4

**Published:** 2019-08-26

**Authors:** Marjory Xavier Rodrigues, Rodrigo Carvalho Bicalho, Nadine Fiani, Svetlana Ferreira Lima, Santiago Peralta

**Affiliations:** 1 Department of Population Medicine and Diagnostic Sciences, Cornell University, Ithaca, NY 14853 United States; 2 Department of Clinical Sciences, Cornell University, Ithaca, NY 14853 United States; 3 Weill Cornell Medicine, Jill Roberts Institute for Research in Inflammatory Bowel Disease, Cornell University, New York City, NY 10021 United States

**Keywords:** Microbiome, Dental diseases

## Abstract

Periodontitis is a common and important health problem in domestic cats. The subgingival microbiota of cats diagnosed with chronic periodontitis (CP), aggressive periodontitis (AP), and feline chronic gingivostomatitis (FCGS) are not well characterized. Thus, the aim of the present study was to characterize and compare the periodontal microbiota of periodontally healthy cats versus cats diagnosed with CP, AP, and FCGS by using next-generation sequencing. In total, 44 domestic cats were enrolled, and 139 subgingival samples were subjected to 16S rRNA gene sequencing to investigate the microbiota composition of each periodontal group evaluated. Our results identified several key genera previously described in periodontal disease (e.g. *Treponema* and *Filifactor*) and in the oral microbiota (e.g. *Moraxella* and *Capnocytophaga*) of healthy cats. Phylogenetic beta diversity analysis showed that the microbiota of periodontally healthy cats were distinguishable from diseased cats. Even though most of the genera known to be associated with periodontal disease were also identified in healthy cats, they were present at significantly lower relative abundance. Remarkably, alpha diversity was found to be higher in the disease groups compared to healthy animals. These results suggest a pathological mechanism involving opportunistic behavior. Our findings corroborate those in the current literature regarding the complexity of the subgingival microbiota of the domestic cat and reveal both differences and similarities among periodontally healthy and diseased cats.

## Introduction

Periodontitis is an inflammatory disease that is highly prevalent in domestic cats^1,2^. It is characterized by loss of periodontal attachment, tooth mobility and eventual tooth loss^2,3^. It causes pain, gingival bleeding, reduced food intake, and may impact overall health^4,5^. Given its prevalence, as well as its local and systemic effects, periodontitis is a serious animal welfare issue that can impact quality of life. The pathogenesis of periodontitis involves the presence of subgingival bacterial plaque that initially leads to gingivitis and marginal tissue inflammation; if not treated it may progress to loss of periodontal attachment^6^.

Periodontitis can be classified as chronic or aggressive^1,3,7,8^. Chronic periodontitis (CP) is the most common form and is characterized by slow progression^1^; the prevalence of CP increases with age^2^. In contrast, aggressive periodontitis (AP) has a higher rate of progression and typically begins during early stages of life^3,9^. Although subgingival bacteria play a crucial role in the disease, determination of the causal pathogen(s) has been inconclusive despite advances in the microbiology of subgingival biofilms in health and in different forms of periodontal disease^9^. In humans, it has been suggested that AP is associated with a mixed and complex microbial flora, with heterogeneity in the types and proportions of microorganisms recovered from individuals with the disease^9^. On the other hand, the supposed association of AP with an increased load of specific periodontal bacteria underscores the importance of bacteria to the disease process, and consequently the importance of microbiologic tests^10^.

Another important disease is feline chronic gingivostomatitis (FCGS), which causes severe pain and distress and is characterized by focal or diffuse chronic inflammation of the gingiva and oral mucosa^11^. FCGS may affect cats of any age, and treatment usually involves lifelong medical therapy or extensive surgery. Many cats fail to respond to treatment, and some are euthanized due to a poor quality of life^12,13^. Even though the etiology of FCGS is unknown, bacteria are thought to play a role in the disease pathogenesis^14–16^. Additionally, cats with FCGS have been shown to develop more severe and extensive forms of periodontitis compared to cats without the disease^17^. However, it is unknown whether the latter is due to underlying differences in the subgingival microbiome compared to cats without FGCS.

Techniques based on DNA sequencing and advanced bioinformatics tools can help unravel the complexity of the subgingival microbiota in periodontal health and disease^10,18^. Recent studies have made use of bacterial 16S rDNA sequencing to describe the feline oral microbial composition^6,14,19,20^. Sturgeon^20^ and colleagues characterized the oral microbiota of healthy cats using next-generation sequencing and found it to be highly diverse, rich, and even. Also, comparisons of the feline subgingival microbiota among healthy, gingivitis, and mild periodontitis cases were recently described based on pyrosequencing^6^. *Porphyromonas* was the most abundant genus in healthy cats, along with *Moraxella* and *Fusobacterium*, whereas *Peptostreptococcaceae* was the most abundant family in gingivitis and mild periodontitis^6^. Dewhirst *et al*.^19^, using bacterial culture and DNA sequencing, showed that for periodontitis as well as for other oral diseases, members of the phyla Spirochaetes and Bacteroidetes are key pathogens^19^, e.g. *Treponema denticola* and *Porphyromonas gingivalis*^21^, respectively. *Pasteurella multocida* subsp. *multocida* was found to be significantly more prevalent in affected cats compared to controls when using culture-dependent and independent methods in one study^14^.

In order to better understand the possible role of the subgingival microbiota in periodontal health and disease in cats, we have applied next-generation sequencing techniques to assess and compare the periodontal microbiota from healthy cats and cats with CP, AP, and FCGS.

## Results

### Initial descriptive data

The intrinsic characteristics of enrolled animals were analyzed. There was no statistically significant difference among disease groups (i.e., AP vs. CP vs. FCGS) regarding sex (male vs female) or breed distribution (domestic vs purebred) using a chi-square test. A Kruskal-Wallis test was used to analyze differences among groups regarding age and body weight. A statistically significant difference was identified among disease groups regarding age (P-value = 0.005). Comparisons for all pairs using the Dunn method showed that cats with AP were significantly younger compared to cats with CP (P-value = 0.0103); significant differences were not found between the other disease groups. No statistically significant differences were found among diseases groups in terms of body weight. Median, minimum and maximum age and body weight are presented in Table 1.

**Table 1:** Median, minimum and maximum age (years) and body weight (kg) of healthy cats and cats affected with affected with chronic periodontitis (CP), aggressive periodontitis (AP), and feline chronic gingivostomatitis (FCGS).

Status	Age (years)			Body Weight (kg)		
	Median	Minimum	Maximum	Median	Minimum	Maximum
Healthy	10.5	2	18	5.2	3.7	9.5
CP	8	3	17	5.5	3.0	7.7
AP	3	2	14	4.8	3.3	6.3
FCGS	4	3	11	3.7	3.2	5.6

### Sequencing results

DNA amplification using barcoded primers and next-generation sequencing of the V4 region of the 16 rRNA gene were completed for all samples collected. The sequencing run was performed using a MiSeq sequencer and the reagent kit v2 (300-cycles) (Illumina, Inc., San Diego, CA). Quality filtered reads were de-multiplexed, and the total number of reads was 5,372,557; the average coverage was 38,651 reads per sample, with a standard deviation of 11,454; the number of reads per sample ranged from 8,476 to 82,373 (median = 36,217); and the median length per read was 301 bases.

### Bacterial distribution based on phylum level

**Figure 1:**
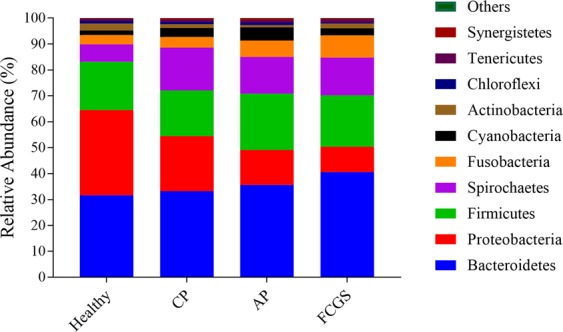
Relative abundance (%) of the most abundant bacterial phyla identified in healthy cats and in cats affected with chronic periodontitis (CP), aggressive periodontitis (AP), and feline chronic gingivostomatitis (FCGS).

The relative distributions of bacteria at the phylum level identified by 16S rDNA gene sequencing are depicted in Fig. 1. The most prevalent bacterial phyla among all cats were Bacteroidetes, Proteobacteria, Firmicutes, Spirochetes, and Fusobacteria. Proteobacteria was the most abundant phylum in the healthy group (mean = 32.88%; median = 34.03%; minimum = 23.57%; maximum = 37.35%), whereas in the disease groups the relative abundance dropped in CP (mean = 21.27%; median = 19.08%; minimum = 6.22%; maximum = 48.96%), AP (mean = 13.42%; median = 12.92%; minimum = 7.62%; maximum = 19.90%), and FCGS (mean = 9.82%; median = 9.08%; minimum = 6.51%; maximum = 12.78%). Bacteroidetes was the most abundant phylum in the disease groups, having a higher relative abundance in the FCGS group (mean = 40.56%; median = 39.38%; minimum = 17.32%; maximum = 59.16%), followed by AP (mean = 35.62%; median = 30.92%; minimum = 21.56%; maximum = 71.37%) and CP (mean = 33.17%; median = 32.69%; minimum = 14.92%; maximum = 58.19%). In addition, Fusobacteria and Spirochaetes were also more abundant in the diseased individuals.

### Response screening analysis

**Figure 2:**
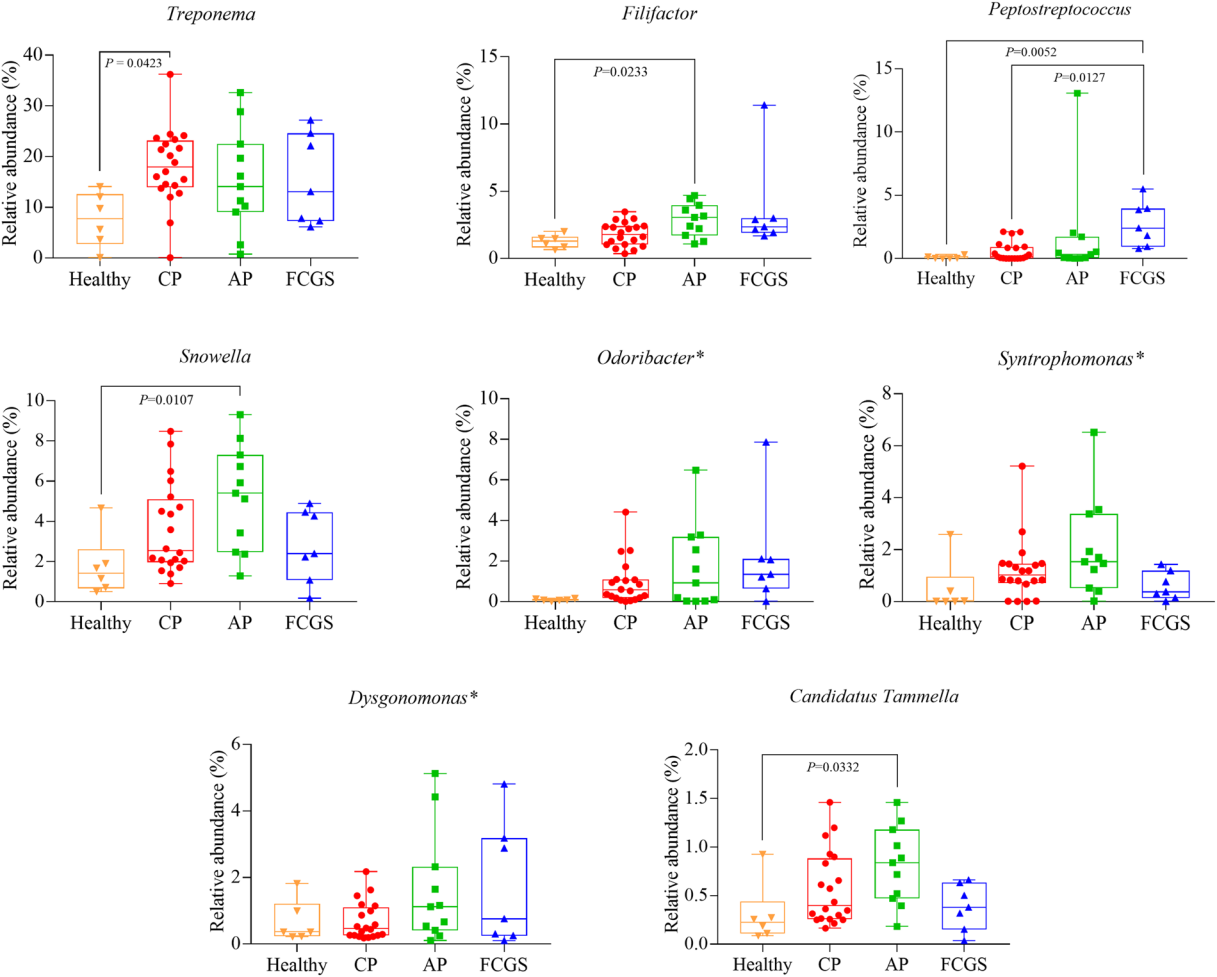
Box and whiskers plots illustrating the median, quartiles, maximum and minimum of relative abundance of bacteria genera found with significantly higher abundance in diseased cats compared with healthy cats. The genera shown were found to be significant based on screening analysis (FDR P-value < 0.1) using the fifty most abundant genera. P-value indicates significant difference between groups using Dunn method (P < 0.05). *P-value not significant using Dunn method. CP = Chronic Periodontitis; AP = Aggressive Periodontitis; FCGS = Feline Chronic Gingivostomatitis.

Each bacterial taxon selected by the screening model was subjected to the Dunn method to examine potential differences across groups. Figure 2 depicts the bacterial genera found to be significantly more abundant in cats with periodontal disease when compared to healthy cats. *Treponema* was the most abundant genus in the disease groups. However, the relative abundance of *Treponema* was significantly higher only in the CP group compared to the healthy group (P = 0.0423). *Snowella* was more abundant in the AP group when compared to the two other disease groups. In addition, the relative abundance of *Filifactor* differed significantly between the healthy and AP groups (P = 0.0233; Fig. 2). *Peptostreptococcus* was significantly different between the healthy group and FCGS (P = 0.0052), and between CP and FCGS (P = 0.0127). *Candidatus Tammella* differed significantly only between the healthy group and AP (P = 0.0332).

**Figure 3:**
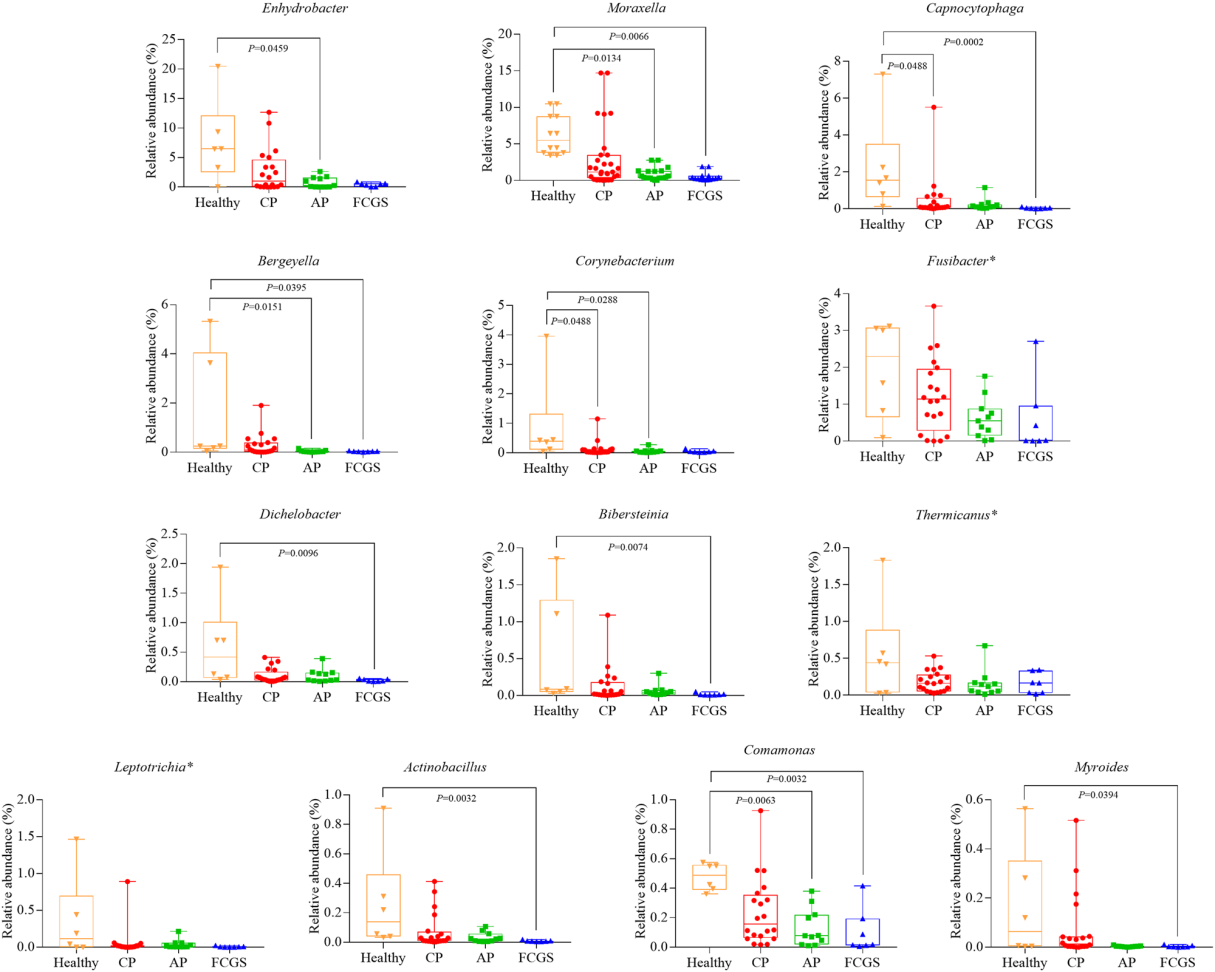
Box and whiskers plots illustrating the median, quartiles, maximum and minimum of relative abundance of bacteria genera found with significantly lower abundance in diseased cats compared with healthy cats. The genera shown were found to be significant based on screening analysis (FDR P-value < 0.1) using the fifty most abundant genera. P-value indicates significant difference between groups using Dunn method (P < 0.05). *P- value not significant using Dunn method. CP = Chronic Periodontitis; AP = Aggressive Periodontitis; FCGS = Feline Chronic Gingivostomatitis.

Bacterial genera identified as being significantly less abundant in the disease groups compared to the healthy group are reported in Fig. 3. *Enhydrobacter*, *Moraxella*, and *Capnocytophaga* were the most abundant genera in healthy cats. When compared to the AP group, the genera *Enhydrobacter* (P = 0.0459), *Moraxella* (P = 0.0134), *Bergeyella* (P = 0.0151), *Corynebacterium* (P = 0.0288), and *Comamonas* (P = 0.0063) were found to be significantly more abundant in healthy cats. Furthermore, *Moraxella* (P = 0.0066), *Capnocytophaga* (P = 0.0002), *Bergeyella* (P = 0.0395), *Dichelobacter* (P = 0.0096), *Bibersteinia* (P = 0.0074), *Actinobacillus* (P = 0.0032), *Comamonas* (P = 0.0032), and *Myroides* (P = 0.0394) were less abundant in FCGS than in the healthy group. When comparisons were performed with the CP group, significant lower relative abundance of *Corynebacterium* (P = 0.0488) and *Capnocytophaga* (P = 0.0488) were identified only when compared to healthy cats.

### Alpha and beta diversity

**Figure 4:**
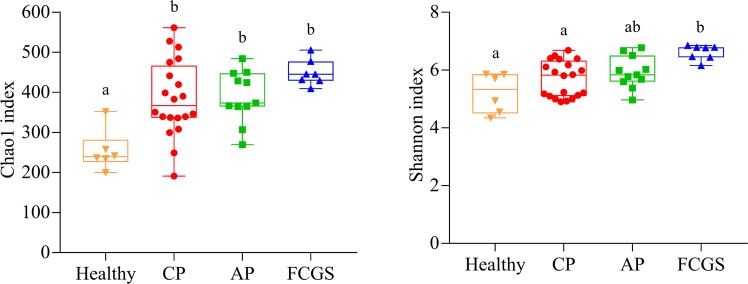
Box and whiskers plots illustrating median, quartiles, maximum and minimum values of the Chao1 richness index and the Shannon diversity index for the different periodontal statuses in cats. Different letters indicate significant differences between periodontal statuses using the Tukey–Kramer test (P-value < 0.05). CP = Chronic Periodontitis; AP = Aggressive Periodontitis; FCGS = Feline Chronic Gingivostomatitis.

The alpha diversity was determined by Chao1 and Shannon indices and is depicted in Fig. 4. The Chao1 richness index (number of species) was significantly higher in diseased cats compared to healthy cats (healthy vs. FCGS, P = 0.002; healthy vs. AP, P = 0.0063; healthy vs. CP, P = 0.0037). No significant differences were found among disease groups. On the other hand, when alpha diversity analysis based on the Shannon diversity index (accounts for microbial distribution; evenness) was performed, a significant difference was found in the disease groups. More specifically, the Shannon index was significantly higher in FCGS group compared to the healthy (P = 0.0004) and CP (P = 0.0041) groups. Furthermore, the microbiota of healthy cats harbored the lowest Shannon diversity index, albeit not significantly different from CP (P = 0.2809) and AP (P = 0.0810). Due to the use of depth of 10,000 reads during the rarefaction process, one sample was eliminated from analysis.

**Figure 5:**
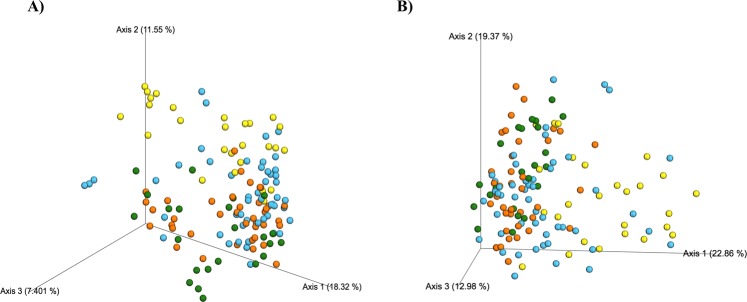
Beta diversity analysis of microbial communities of subgingival samples from healthy cats (yellow points) and cats affected with chronic periodontitis (CP; blue points), aggressive periodontitis (AP; orange points), and feline chronic gingivostomatitis (FCGS; green points). (**A**) Principal coordinate analysis (PCoA) based on unweighted (OTU presence/absence) UniFrac distances metrics (P-value = 0.001; R-squared = 0.103); (**B**) PCoA based on weighted (OTU abundances) UniFrac distance metrics (P-value = 0.001; R-squared = 0.13). In the parentheses is the variance explained by each PCoA and each point corresponds to a microbial community colored according to type of sample (healthy, AP, CP, and FCGS).

To assess microbial community similarities among the different groups, beta diversity was calculated by using UniFrac distance metrics. Regardless of the UniFrac metric used (unweighted: OTU presence/absence; qualitative or weighted: OTU abundance, quantitative) microbial communities significantly clustered by periodontal status (unweighted UniFrac: P = 0.001; weighted UniFrac: P = 0.001, Fig. 5). More detailed analyses were performed to evaluate pair microbiota composition similarities and are depicted in Table 2.

**Table 2:** Comparison of subgingival microbial community composition between healthy and disease groups on weighted and unweighted UniFrac distances.

UniFrac	Mann-Whitney P-value	Holm–Bonferroni P-value
Unweighted		
Healthy vs. AP	<0.0001	<0.0001
Healthy vs. CP	<0.0001	<0.0001
Healthy vs. FCGS	0.0119	<0.0001
AP vs. CP	0.4340	<0.0001
AP vs. FCGS	0.0155	<0.0001
CP vs. FCGS	0.0031	<0.0001
Weighted		
Healthy vs. AP	<0.0001	<0.0001
Healthy vs. CP	<0.0001	<0.0001
Healthy vs. FCGS	<0.0001	<0.0001
AP vs. CP	0.0001	<0.0001
AP vs. FCGS	0.0692	<0.0001
CP vs. FCGS	0.6950	<0.0001

CP = Chronic Periodontitis; AP = Aggressive Periodontitis; FCGS = Feline Chronic Gingivostomatitis.

## Discussion

To better understand the subgingival microbiota of CP, AP, FCGS and periodontally healthy domestic cats, we used a 16S target sequencing approach. Significantly different relative abundances were identified among groups, both between healthy and diseased cats and among diseases. As was expected, genera with pathogenic species (e.g. *Treponema* and *Filifactor*) were detected as significant in the disease groups, as were genera known to be constituents of the oral microbiota of healthy cats (e.g. *Moraxella* and *Capnocytophaga*). However, we also identified significant genera which, to our knowledge, have not yet been associated with periodontal disease in cats, thus demanding further in-depth studies. Additionally, we found higher alpha diversity in the disease groups compared to the healthy cats. Notably, regardless of the method used to evaluate the beta diversity, healthy cats were found to harbor a microbial community that differed from that detected in diseased cats. Therefore, this study corroborated previous data and revealed new insights into feline periodontal disease and its different clinical forms and presentations.

The most represented phyla were Bacteroidetes, Proteobacteria, Firmicutes, Spirochetes and Fusobacteria, regardless of periodontal status. The occurrence and dominance of these phyla is consistent with previous studies^19,20^. Of note, we identified a lower abundance of Fusobacteria in healthy cats, in contrast with a previous study^6^. We also found the phylum Spirochaetes to have a higher abundance in periodontally diseased cats compared to healthy, in agreement with a study by Harris *et al*.^6^. Several studies have reported that Spirochaetes are associated with periodontitis in numerous species, including humans and dogs^22–27^, suggesting that the bacteria’s possible pathogenic role is conserved regardless of the host. Spirochaetes includes the genus *Treponema*, which we found to be one of the most prevalent taxa and to have a significantly higher relative abundance in the CP group compared to the healthy group. A similar result was reported by Harris *et al*.^6^ in an investigation of the subgingival microbiota in healthy cats and cats with gingivitis and mild periodontitis. This is not surprising considering that *Treponema* spp. carry conserved and unique virulence factors that promote survival and pathogenicity^24^. The phylum Bacteroidetes comprises *Porphyromonas* spp., which are also highly prevalent in cats with periodontitis^28,29^. A previous study showed that *Porphyromonas gulae* is the most relevant pathogen in periodontal diseases in cats^28^. In humans, *Porphyromonas gingivalis* is the major pathogen which contributes to CP^30^. Norris *et al*.^29^ concluded that *Porphyromonas gingivalis* acts as an opportunistic periodontal pathogen in at least humans and cats.

*Treponema*, *Snowella* and *Filifactor* were the most abundant genera, with relative abundances significantly higher in the disease groups compared to the healthy group. These results are consistent with the ecological plaque hypothesis^31^, which proposes a model whereby disease-associated bacteria comprise a minor portion of the subgingival flora of healthy tissues but increase significantly as periodontal disease progresses. To our knowledge, subgingival *Snowella and Candidatus Tammella* have not been previously reported in cats. In the present study, significantly higher abundance of these genera was found in AP, which suggests a role for these genera in the pathogenesis of this form of the disease. Vieira Colombo *et al*.^32^ stated that the ecological diversity of the periodontal environment may support colonization of species not typically considered as members of the oral microbiota; thus, our findings should not be used as proof of pathogenicity. Further studies will be necessary to elucidate the possible pathogenic role of these two genera in AP.

*Filifactor* and *Peptostreptococcus* belong to the *Peptostreptococcaceae* family. This family was identified as the most abundant in gingivitis and mild periodontitis in a previous study in cats^6^. Based on differences in relative abundance across disease groups, our findings suggest that these two taxa might play a role in periodontitis in cats with FCGS.

In contrast, *Enhydrobacter* was the most abundant bacterial genus detected in healthy cats, with a significantly lower relative abundance in the disease groups compared to healthy cats, followed by *Moraxella* and *Capnocytophaga*. *Moraxella* and *Capnocytophaga* have been highlighted as playing a significant role in feline periodontal health^6,14,20^. Interestingly, *Enhydrobacter*, classified in the phylum Proteobacteria, was reported to have a significantly higher relative abundance in human non-smokers compared to smokers in a study of severe chronic periodontitis^33^. *Enhydrobacter* was also identified as being highly prevalent in dogs with healthy periodontium^34^, which underscores significant differences in the subgingival microbiome associated with disease across species.

Genera such as *Treponema*, *Porphyromonas*, and *Peptostreptococcus*, which are known to be associated with periodontal disease, were not found solely in any one disease or shared only among disease groups but were also identified in the microbiome of healthy cats. This finding is consistent with the ecological plaque hypothesis in which the subgingival environment selects the microbial flora and drives the transformation from health to disease^31^. As a putative mechanism, nonspecific plaque accumulation leads to inflammation of gingival tissues, and the concurrent changes in the local environment favor Gram-negative and proteolytic bacteria^31^. Bartold & Van Dyke^31^ hypothesized that it is the inflammation within tissues that drives the microbial changes and not the other way around. Thus, biofilm progression from supragingival to subgingival sites drives a shift from an aerobic to an anaerobic environment, restricting the growth of the early Gram-positive facultative aerobes and favoring mainly Gram-negative anaerobes^35^. Furthermore, many Gram-negative anaerobes require amino acids or small peptides for growth, and the gingival crevicular fluid, which is enriched with peptides, selects for these bacteria^31^. Thus, periodontal disease is associated with overgrowth of specific microorganisms as a consequence of microenvironmental changes^31^. In agreement, based on our response screening analysis, the majority of genera we identified as being more abundant in disease categories were Gram-negative and anaerobic, i.e. *Treponema*, *Odoribacter*, *Syntrophomonas*, *Candidatus Tammella*, and *Dysgonomonas*; the respective information was not found for the *Snowella* genus.

Microbial community analysis using phylogenetic information showed that the environments (healthy, CP, AP, and FCGS) were distinguishable. When comparisons were used to evaluate differences between groups, significant Unifrac distances were identified between healthy and disease groups. Accordingly, Chao1 and Shannon indices were higher in diseased cats. A previous study on periodontitis in humans using 16S pyrosequencing revealed that community diversity was higher in disease sites than in healthy sites^22^. In contrast to our findings, Dolieslager *et al*.^14^ concluded that the oral flora in cats with FCGS is less diverse than in healthy cats.

In summary, our study corroborates findings in the literature regarding the complexity of the subgingival microbiome of the domestic cat and demonstrates differences and/or similarities based on periodontal status. We found higher bacterial diversity in the microbiomes of diseased sites compared to healthy sites, highlighting the important role played by bacterial biofilms in ecologically unstable tissue environments. Also, a high prevalence and significance of bacteria previously described as pathogens was demonstrated in disease groups. Additionally, beta diversity showed dissimilar bacterial composition between the groups studied. Further studies should be conducted with advanced DNA sequencing tools to improve the understanding of these diseases in cats based on genes and microorganisms from subgingival samples.

## Methods

### Ethics statement

Experimental protocols using cats were reviewed and approved by Cornell University Institutional Animal Care and Use Committee (protocol number 2015-0117). Informed consent for study participation was obtained from all cat owners prior to experimental sampling. The methods were carried out in accordance with the approved guidelines.

### Case definition

Periodontal status was assessed by a board-certified veterinary dentist based on periodontal probing and full-mouth radiographic findings. The Silness-Löe index was used to determine the gingival index during periodontal examination^36^. Briefly, a gingival index of 0 was assigned when there was no visible gingival inflammation or bleeding upon periodontal probing; a gingival index of 1 was assigned when there was gingival erythema/edema but no bleeding upon probing; a gingival index of 2 was assigned when there was gingival erythema/edema and mild gingival bleeding upon probing; a gingival index of 3 was assigned when there was gingival erythema/edema and spontaneous bleeding without probing. Cats with periodontal probing depths <1 mm, no radiographic evidence of alveolar bone loss, and a gingival index of 1 or less on every tooth, were considered healthy controls. For the purposes of this study, CP was diagnosed when the cat had clinical and/or radiographic evidence of clinical attachment loss in 1 or more teeth regardless of the severity, had a gingival index of 2 or less in at least 70% of the teeth present, and had no signs of inflammation or ulceration beyond the mucogingival junction. AP was diagnosed when the cat had a gingival index of 3 affecting 30% or more of the teeth present; had more than 50% of clinical attachment loss affecting 30% or more of the teeth present; and had no signs of inflammation or ulceration beyond the mucogingival junction or at the caudal oral mucosa. A diagnosis of FCGS was made based on clinical examination findings including ulcerative or ulcero-proliferative lesions of the caudal oral mucosa, as previously described^37^.

For the purposes of this study, when applicable, the stage of progression of periodontitis of sampled teeth was determined based on the following criteria: mild periodontitis, if there was clinical or radiographic evidence of less than 25% of attachment loss; moderate, if there was clinical or radiographic evidence of 25–50% of attachment loss; or severe if there was clinical or radiographic evidence of more than 50% of attachment loss.

Animals that had received systemic antibiotics during the previous 4 weeks were excluded. Only cases in which full-mouth radiographs and periodontal probing and charting were performed were included in this study.

### Sample collection

The sampled population consisted of client-owned domestic cats originally presented to the Cornell University Hospital for Animals (CUHA) Dentistry and Oral Surgery Service for diagnosis and treatment of periodontal disease and/or FCGS. In total, 139 subgingival samples, up to 7 samples per animal, were obtained from 44 cats, each individual was classified in only one category. The sex distribution of sampled animals was 23 males (52.3%) and 21 (47.7%) females. The breed distribution was 39 domestic and 5 purebred cats (1 Bengal, 2 Persian, and 2 Siamese). Out of the 139 samples, 27 samples came from 6 healthy control animals, 50 samples from 20 cats with CP, 39 samples from 11 cats with AP, and 23 samples from 7 cats with FCGS. Of the samples collected from the CP group, 31 (62%) were from sites previously diagnosed with mild periodontitis, 15 (30%) were from sites diagnosed with moderate periodontitis, and 4 (8%) were from sites with severe periodontitis. In the AP group, 21 (53.8%) samples were collected from sites diagnosed with mild periodontitis, 16 (41%) samples were from sites with moderate periodontitis, and 2 (5.1%) samples were collected from sites diagnosed with severe periodontitis. For the cats with FCGS, 10 (43.5%) samples were collected from sites diagnosed with mild periodontitis, and 9 (39.1%) and 4 (17.4%) samples were collected from sites diagnosed with moderate and severe periodontitis, respectively.

Only canine teeth and carnassial teeth (maxillary fourth premolar and/or mandibular first molar teeth) were sampled. The sample side (i.e. right vs. left) was selected randomly. In the disease groups (i.e. AP, CP and FCGS), only samples from teeth with periodontitis were included for analysis. Of the 27 samples collected from healthy cats, 13 (48.1%) were obtained from canine teeth and 14 (51.9%) from carnassial teeth. Of the 50 samples obtained from cats with CP, 24 (48%) were obtained from canine teeth and 26 from carnassial teeth. Of the 39 samples obtained from cats with AP, 17 (43.6%) were obtained from canine teeth and 22 (56.4%) from carnassial teeth. Of the 23 samples obtained from cats with FCGS, 6 (26.1%) were obtained from canine teeth and 17 (73.9%) from carnassial teeth, mainly because canine teeth in cats with FCGS often did not have clinical or radiographic signs of periodontitis.

Subgingival samples were obtained prior to any instrumentation or disinfection, or systemic antibiotic administration. In the present study, the established technique of using a sterile endodontic paper point^38^ was used for collection of subgingival samples. Briefly, cats were clinically evaluated and sampled under general anesthesia using standard-of-care protocols supervised by a board-certified veterinary anesthesiologist. Each sterile endodontic paper point (absorbent paper points - Coarse, Meta^®^ Dental Corp., Glendale, NY) was gently inserted into the sulcular area and gently rubbed along at least half of the circumference of individual teeth. Samples were aseptically collected, labeled, and stored individually in 1.5 ml sterile polypropylene microcentrifuge tubes, placed on ice, transported to the laboratory within 4 h, and then frozen at –80 °C.

### DNA extraction, DNA amplification and next-generation sequencing of the bacterial 16S rRNA gene

DNA was extracted from paper points by adding 1.0 mL of UltraPure^TM^ distilled water (DNAse and RNAse free, Invitrogen, Grand Island, NY) into a 1.5 mL microcentrifuge tube containing the paper point, which was placed in a vortex mixer (Fisher Scientific, Hampton, NH) and vortexed/washed for 10 minutes. Paper points were removed from the microcentrifuge tubes and the remaining liquid was submitted to centrifugation for 5 min at 13,000 rpm at room temperature. The pellet obtained was used for DNA extraction, which was performed using a DNeasy^®^ PowerFood^®^ Microbial Kit (Qiagen, Hilden, Germany) following the manufacturer’s instructions.

Amplification of the V4 hypervariable region of the bacterial 16S rDNA gene for each sample was carried out by polymerase chain reaction (PCR) using primers 515F and 806R according to a previously optimized method for the Illumina MiSeq platform^39^, all DNA samples were amplified using different 12-bp error-correcting Golay barcodes for 16S rRNA gene PCR (http://www.earthmicrobiome.org)^40^. Amplifications were performed using 10 µM of each primer, EconoTaq Plus Green 1x Master Mix (Lucigen^®^, Middleton, WI), 10 ng–100 ng of individual metagenomic DNA and UltraPure^TM^ distilled water (DNAse and RNAse free, Invitrogen, Grand Island, NY) to bring the final reaction volume to 25 µL; each DNA sample was amplified in triplicate and in all PCR plates a blank (no DNA added) was included. Also, a negative control (new paper point, no DNA sample added) and a positive control (*Escherichia coli*, pure culture) were included. PCR conditions were: initial denaturing at 94 °C for 3 min; 35 cycles of 94 °C for 45 s, 50 °C for 1 min, and 72 °C for 90 s; and final elongation at 72 °C for 10 min. Pooled replicates were loaded in agarose gels (1.2%, wt/vol) stained with 0.5 mg/ml ethidium bromide; electrophoresis was completed and the presence of amplicons was verified.

Amplified DNA was purified using a Gel/PCR Fragments Extraction Kit (IBI Scientific, Peosta, IA, USA) and the concentration of each purified DNA sample was determined using a NanoDrop ND-1000 spectrophotometer (NanoDrop Technologies, Rockland, DE, USA). Aliquots of all samples were standardized to the same concentration and pooled for sequencing on the Illumina MiSeq platform (Illumina^®^ Inc., San Diego, CA). Final equimolar libraries were sequenced using the MiSeq Reagent Kit V2–300 cycles (Illumina^®^ Inc., San Diego, CA).

### Bioinformatics

All 16S rRNA gene sequences generated were processed through the open-source pipeline Quantitative Insights into Microbial Ecology (QIIME) version 1.7.0-dev^39^. Quality filter was applied for sequences using established guidelines^41^; in addition, UCLUST^42^ was applied to bin sequences into operational taxonomic units (OTUs) based on 97% identity against the Greengenes reference database^43^ (May 2013 release). Using USEARCH^42^, low abundance clusters were filtered, and chimeric sequences removed. Representative sequences for each OTU were compared against the Greengenes database for taxonomy assignment, and only full-length, high-quality reads (−r = 0) were used for data analysis. Phylogenetic trees were generated from the filtered alignment using FastTree^44^. Shannon diversity index and Chao1 richness index output were generated by the QIIME pipeline; prior to index estimation, the sample library was rarefied to an equal depth of 10,000 sequences using QIIME. To determine how taxa were related within and between subgingival microbiota of healthy cats and cats diagnosed with CP, AP, FCGS, both unweighted and weighted UniFrac distance metrics were generated in QIIME^45^. To account for uneven sequencing depth across samples, all sample libraries were rarefied to an equal depth of 10,000 sequences before estimating the unweighted and weighted UniFrac. Also, phylum- and genus-level OTU tables were generated using the MiSeq Reporter Metagenomics Workflow, which is based on the Greengenes database (http://greengenes.lbl.gov/). The output from this workflow is a classification of reads at multiple taxonomic levels (kingdom, phylum, class, order, family, genus, and species).

### Statistical analysis

Descriptive statistical analysis was completed in JMP Pro 11 (SAS Institute Inc., Cary, NC) using the chi-square test of independence to evaluate whether cat gender and breed differed significantly between groups. The Kruskal-Wallis test and the Dunn method were used to determine whether there were statistically significant differences between groups regarding age and body weight; both tests were completed using JMP Pro 11.

The OTU tables obtained from bioinformatics analyses were used to describe the relative abundances of bacterial phyla and genera within the healthy and diseased cats. Each value obtained indicates the percentage relative frequency of reads with 16S rRNA genes annotated to the indicated taxonomic level. The arithmetic mean of the relative abundance of each OTU was calculated for each individual cat, a single parameter.

The distribution of bacterial phyla across the groups studied was presented according to the average of relative abundance calculated by animal enrolled as described. The microbial profile of healthy and disease groups for the most abundant phyla was fitted using GraphPad Prism 7 (GraphPad Software LLC, La Jolla, CA).

Response screening analysis was performed in JMP Pro 11 to determine which bacterial taxa are most associated or most important to the different periodontal status groups. The relative abundances of each genus found to have a False Discovery Rate (FDR) of <0.1 were illustrated by box-whiskers plots. A nonparametric comparison for all pairs using the Dunn method were performed using JMP Pro 11 to evaluate statistical differences among the different groups evaluated.

Chao 1 richness and Shannon diversity indexes were calculated using QIIME. These diversity indexes were compared within all periodontal statuses using ANOVA in JMP Pro 11, and the Tukey-Kramer test (P < 0.05) was used to adjust for multiple comparisons. Box-whiskers plots were built using GraphPad Prism 7 (GraphPad Software LLC, La Jolla, CA).

Differences between microbial communities (beta-diversity) based on phylogenetic information visualized on the PCoA plots were calculated by permutational multivariate analysis of variance (PERMANOVA) with 999 permutations^46^ by QIIME (http://qiime.org/scripts/compare_categories.html). Principal coordinates were computed from the calculated UniFrac distance matrixes to compress dimensionality into three-dimensional principal coordinate analysis (PCoA) plots created by the “beta_diversity_through_plots.py” script in QIIME and visualized by EMPeror^47^. For calculation of pairwise ecological distances, the Mann-Whitney test followed by Holm–Bonferroni multiple comparison correction were used.

## Data Availability

The datasets generated during and/or analyzed during the current study are available from the corresponding author on reasonable request.
